# Nest architecture and male aggression drive sexual isolation between three-spined stickleback ecotypes

**DOI:** 10.1186/s40851-026-00263-w

**Published:** 2026-02-12

**Authors:** Carl Smith, Rowena Spence, Eliza Szczerkowska-Majchrzak, Dawid Żegota, Grzegorz Zięba

**Affiliations:** 1 Department of Ecology and Vertebrate Zoology, University of Łódź, Banacha 12/16, Łódź, 90-237 Poland; 2 Institute of Vertebrate Biology, Czech Academy of Sciences, Květná 8, Brno, 603 00 Czech Republic

**Keywords:** Assortative mating, Courtship, Fertilisation, Mate choice, Reproduction, Sexual selection

## Abstract

To understand how sexual isolation contributes to the evolution of reproductive barriers, we examined the pre- and post-zygotic reproductive barriers between ecotypes of the three-spined stickleback (*Gasterosteus aculeatus*) from North Uist, where the species has undergone an adaptive radiation. Using an experimental design that separated the effects of ecotype, population of origin, and body size, we identified a pre-zygotic barrier attributed solely to ecotype; pairings within ecotypes were more likely to result in mating than those between ecotypes, regardless of population origin or relative body size of males and females. This effect was associated with reproductive incompatibilities linked to the failure of armoured females to enter the unusually small nests of armourless males, along with high levels of aggression from armoured males directed at armourless females. In vitro crosses did not reveal post-zygotic reproductive barriers associated with ecotype or population of origin. These findings indicate that sexual isolation, linked primarily to male traits and involving the coupling of barrier effects, is a significant obstacle to gene flow among the ecotypes, supporting an emerging view that pre-zygotic sexual isolation tends to evolve rapidly and at an early stage during ecological divergence.

**Supplementary information:**

The online version contains supplementary material available at 10.1186/s40851-026-00263-w.

## Introduction

The extent to which gene flow between populations is constrained serves as a measure of reproductive isolation and represents a fundamental aspect of speciation [1]. Speciation typically involves the gradual accumulation of reproductive isolation, starting with partial barriers that allow limited gene flow and potentially culminating in complete isolation [2]. However, the mechanisms, relative significance, and temporal emergence of barriers to gene flow between populations are often unclear [3]. Speciation is not an inevitable consequence of reproductive isolation, and with restricted gene flow, partially isolated populations that do not fully evolve into separate species may arise instead [2]. Divergence can also reverse when environmental conditions change, shifting selective pressures and eroding reproductive isolation [4].

Barriers to gene flow can be broadly categorised into pre- and post-zygotic [5]. Pre-zygotic barriers occur before fertilisation and include spatial, temporal, behavioural, and mechanical barriers, as well as gametic incompatibility, sperm competition failure, and cryptic female choice [3, 5]. Post-zygotic barriers occur after fertilisation and include hybrid inviability, hybrid sterility, and hybrid breakdown [6]. Pre-zygotic barriers may emerge early in the process of divergence, with post-zygotic isolation evolving later and more gradually and further reinforcing speciation [3]. However, pre-zygotic barriers need not always precede post-zygotic isolation, and the evolution of post-zygotic barriers may correspond with or precede pre-zygotic barriers, especially if divergence occurs in allopatry [6, 7]. The sequence and strength of reproductive isolation mechanisms vary across ecological and evolutionary contexts, shaping the evolution of barriers to gene flow. Ecological factors, such as habitat variation or resource competition, often drive early pre-zygotic barriers, like ecological or behavioural isolation. For example, cichlid fish readily diverge into distinct feeding niches [8], and plants adapt to contrasting soil types [9]. Evolutionary factors—genetic architecture, divergence time, and population structure—influence whether pre-zygotic barriers (e.g. mate choice or temporal isolation) or post-zygotic barriers (e.g. hybrid sterility) predominate [3, 5]. For instance, allopatric speciation arises from geographic isolation, whereas sympatric speciation typically results from ecological or sexual selection [10]. In hybrid zones, reinforcement can further strengthen pre-zygotic barriers to reduce costly hybridisation [11]. Ultimately, the interplay of environment, genetic factors, and species-specific features of mating systems will determine the trajectory and dominance of reproductive isolation barriers.

Insights into the evolution of reproductive isolation can be gleaned from studies of recently diverged groups. At an early stage of divergence, when reproductive isolation is incomplete, the order of reproductive barrier evolution and disparities in the importance of different barriers are likely more apparent because insufficient time has passed for significant genetic, ecological, or behavioural differences to accumulate [12]. As time progresses, selection, drift, or reinforcement will strengthen these barriers, and when multiple barriers accumulate, it can be more difficult to identify the order of barrier evolution and identify whether the current isolating mechanisms contributed to divergence or accumulated after initial divergence.

A key question in speciation research is to identify what biological barriers limit gene flow and how these barriers originate [5]. Pre-mating sexual isolation, defined as the failure of individuals from two populations to mate with one another due to differences in mating behaviour, courtship signals, or sexual preferences, even when other barriers are absent, serves as a key barrier to gene exchange that operates before other isolating mechanisms take effect [5]. While female mate choice is widely recognised as a predominant factor in sexual isolation among animal species, male behaviour may also contribute to the reinforcement of mating barriers [13]. In species in which males engage in nest-building or parental care, male rather than female traits may emerge as the primary reproductive barriers to gene flow.

Here, we examine the mechanisms of sexual isolation between ecotypes of the three-spined stickleback (*Gasterosteus aculeatus*) from a recent adaptive radiation on the island of North Uist in the Scottish Outer Hebrides. North Uist sticklebacks are ideal for investigating barriers to gene flow due to their recent radiation, the large number of isolated populations on the island, and the unusual level of phenotypic, behavioural and genetic diversity among populations over a small geographic area [14–16]. We focus particularly on features of the mating system, including male territoriality, courtship and nest-building, which are recognised to function as reproductive barriers in other taxa [17–19].

In addition to the globally ubiquitous low-plated, completely-plated and partially-plated armour ecotypes [20], populations of three-spined sticklebacks on North Uist exhibit striking variation in protective armour, comprising variation in the size and thickness of lateral bony plates, dorsal spines, pelvic spines, and pelvic girdle [21–23] (Fig. S1), as well as unusual variation in body size [16, 24] and nuptial colouration [25]. Many populations possess no lateral plates, dorsal or pelvic spines, or pelvic girdle [14, 15], and are exclusively associated with acidic peat lochs [16, 22, 26]. In some cases, individuals expressing this ‘armourless’ ecotype may have 1–2 vestigial plates or dorsal spines, but these probably offer no functional protection. Populations of three-spined sticklebacks on North Uist are thought to have been founded from a marine ancestral population approximately 10,000 years ago, resulting in several hundred relatively isolated freshwater populations. Although generally considered independent colonisations, some genomic and geological evidence suggests possible multiple colonisation waves, with weak clustering among some populations in phylogenetic analyses [27]. Phenotypic divergence from the marine ancestor is mirrored by genomic divergence, with more derived phenotypes associated with alleles that are rare in the ancestral marine population [27].

To examine reproductive isolation between recently diverged North Uist ecotypes, we conducted experimental mate choice trials, combined with in vitro fertilisations, to identify reproductive barriers between allopatric low-plated (armoured) and armourless ecotypes, selected for their extreme divergence in armour and ecology. Armourless fish lacked pelvic spines, a pelvic girdle, lateral plates (or possessed just 1–2 vestigial plates), and dorsal spines (or 1–2 vestigial spines). In contrast, the armoured ecotype possessed robust pelvic spines and pelvic girdle, 4–8 lateral plates, and three fully developed dorsal spines (Fig. S1).

To disentangle ecotype-specific effects from those of population of origin and body size, we selected fish with overlapping body sizes among populations and incorporated all factors into a balanced experimental design pairing individuals from two populations per ecotype. All fish used in the study belonged to the same 1+ age class and had not previously completed a breeding season and, consequently, did not differ in reproductive experience. This approach represents a key strength of the study design by allowing isolation of ecotype as the primary driver of any observed reproductive barriers. We aimed to establish whether mating barriers exist between ecotypes or populations at the pre-zygotic (measured as failure to spawn), post-mating pre-zygotic (measured by failure to fertilise eggs), or post-zygotic stage (measured as failure of eggs to survive to hatching), and whether these barriers arose predominantly from divergence in male or female traits. To assess how nesting male three-spined sticklebacks respond to a different ecotype, we also investigated their reaction to female nine-spined sticklebacks (*Pungitius pungitius*). The two species are estimated to have diverged 26.6 Ma [28], but are superficially similar in morphology and ecology [29].

We tested the predictions that: 1. armourless males build smaller nests than armoured males, limiting the ability of armoured females to enter the nests of armourless males to spawn; 2. armoured males direct more aggression toward armourless females (and nine-spined stickleback females) than toward armoured females, which limits the spawning success of armourless females in heterospecific pairings, 3. an outcome of these mating asymmetries is that within-ecotype pairings will have significantly higher spawning probability than between-ecotype pairings, with ecotype effect independent of population of origin or body size. Finally, we predicted: 4. no post-mating or post-zygotic barriers to reproduction based on in vitro fertilisations.

## Materials and methods

### Sampling sites, fish collection and husbandry

**Fig. 1:**
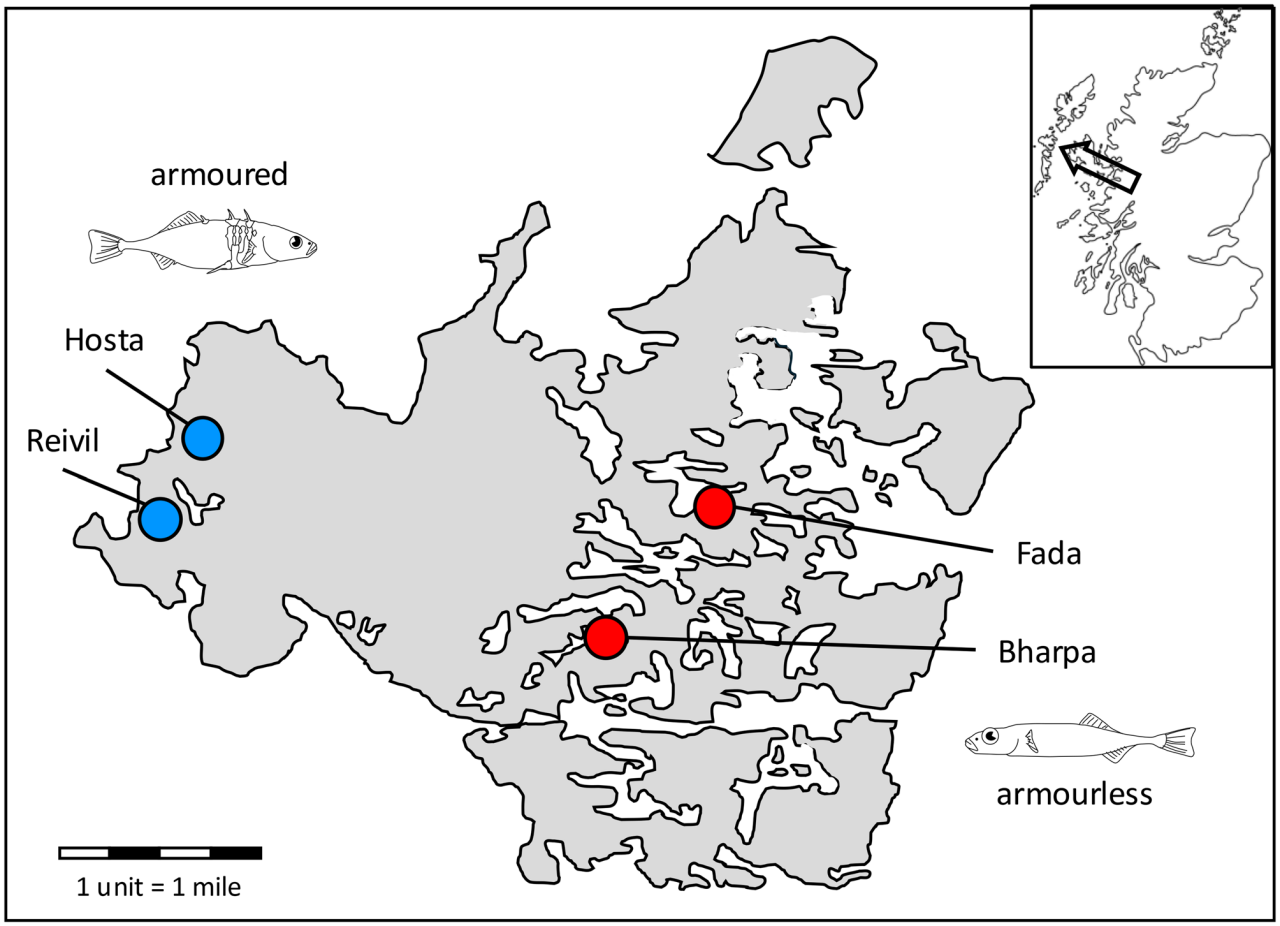
Map of sampling sites on North Uist. Lochs Hosta and Reivil have populations of low-plated armoured three-spined sticklebacks, and Lochs Bharpa and Fada have armourless populations. The inset shows the position of North Uist in relation to mainland Scotland

Three-spined sticklebacks were collected with hand nets from four populations on North Uist in April 2025. To disentangle ecotype-specific effects from those of population of origin, two populations (Lochs Fada and Bharpa) comprised an armour-reduced ecotype from peat-stained lochs of low pH (hereafter termed ‘armourless’), and the other two (Lochs Reivil and Hosta) comprised low-plated ecotypes (‘armoured’) from calcium-rich *machair* lochs (Table 1, Fig. 1). These populations are unconnected, and the populations in them are genetically discrete [27].

**Table 1:** Populations of three-spined sticklebacks used in the study

population	map reference	ecotype	loch type	pH	**area (km**^**2**^)
Loch Bharpa	57.572812, −7.295417	armourless	peat loch	6.2	0.53
Loch Fada	57.614524, −7.223573	armourless	peat loch	6.1	1.93
Loch Hosta	57.627345, −7.490477	armoured	*machair* loch	7.8	0.33
Loch Reivil	57.611216, −7.513549	armoured	*machair* loch	8.7	0.06

After capture, fish were transported in loch water to an aquarium facility with ambient temperature (12–15 °C) and natural daylight. Males were housed individually in plastic aquaria measuring 350 mm (length) ×250 mm (width) ×200 mm (depth). Males expressed the full range of reproductive behaviours in these aquaria, and aquaria of this size are routinely used in studies of male three-spined stickleback nest-building [30], territoriality [31], and parental care [32]. The aquaria were continuously aerated and filtered, and were furnished with artificial vegetation to serve as refuges [33] and landmarks for orientation [34]. Each aquarium had a 10 mm layer of washed sand from the loch of origin, and was filled with 150 mm of water from the same loch. Males were provided with material to construct a nest comprising three hundred 70 mm strands of black polyester thread. Preliminary studies demonstrated that males from each of the four populations readily constructed nests under these conditions, and the same has been demonstrated in other studies [30, 35]. To encourage nest building, males were presented with a gravid female from their population of origin in a square-sided glass jar (95 ×95 × 95 mm) daily for 10 min.

Females from the same population were housed together in 80-litre tanks provided with artificial vegetation as refuges and continuous aeration and filtration in water from their loch of origin. All fish were fed daily *ad libitum* with bloodworm (*Chironomus* spp.) and *Artemia* nauplii. Water quality in aquaria and tanks was monitored for ammonia, nitrite and nitrate using aquarium test kits (NT Labs, Tonbridge, UK). Dissolved oxygen was not measured, as continuous aeration readily maintains near saturation levels ( > 8 mg L^−1^) at the stocking densities used.

### Mating trials

We used no-choice mating trials since this design works well with three-spined sticklebacks by circumventing aggression among males. Territorial male three-spined sticklebacks also steal nest material from neighbouring males and disrupt courtship and spawning [36, 37], making choice trials impractical in this study. No-choice mating trials measure realised spawning success, rather than mate preference, which requires simultaneous choice among multiple partners. We recognise that a no-choice design may overestimate behavioural interference (such as elevated aggression by the male) and also cannot distinguish female mate choice from male-driven barriers or mechanical incompatibilities.

The experimental design permitted us to separate the effects of ecotype, population of origin and body size. Thus, pairings were made of sticklebacks of two discrete ecotypes from four populations. Because the mean body size of the two ecotypes differed [16], there was a risk of confounding body size with population and ecotype. To avoid the risk of collinearity, experimental fish were selected to ensure body sizes overlapped among ecotypes and populations. Males and females from every population were paired in every possible combination, generating 16 pairings in a single complete replicate. Five full replicates of every pairing were conducted over 25 days during April and May 2025 for Lochs Fada, Hosta and Reivil. Male three-spined sticklebacks from Loch Bharpa proved to be timid in experimental aquaria and took longer to build nests than males from the other three populations. Consequently, only four replicates with this population were completed. To maintain the balance of the study design with respect to ecotype, an additional replicate with a male from Loch Fada was undertaken. Thus, the study was unbalanced among populations but balanced between ecotypes, yielding a total of 80 male-female pairings (Table 2).

**Table 2:** Number of mate choice trials conducted for each combination of three-spined stickleback sex (female, male), ecotype (armoured, armourless), and population (Bharpa, Fada, Hosta, Reivil). Figures in parentheses indicate the number of spawnings in each pairing

Sex			Female			
	Ecotype		armourless		armoured	
		Population	Bharpa	Fada	Hosta	Reivil
Male	armourless	Bharpa	4(2)	4(1)	4(0)	4(0)
		Fada	6(2)	6(5)	6(0)	6(0)
	armoured	Hosta	5(0)	5(0)	5(5)	5(5)
		Reivil	5(0)	5(1)	5(4)	5(2)

Once a male had completed the construction of a nest (usually within 4 days); obvious by the male passing through the nest when presented with a gravid stimulus female from the male’s population of origin [38], a gravid female of a pre-selected ecotype and population was transferred to a 30 × 20 ×20 plastic aquarium containing 10 L of water from the male’s loch of origin for 1 h to limit stress associated with the change in pH [39]. After 1 h, the female was introduced into the male’s aquarium in a glass box measuring 95 (depth) ×95 (width) ×95 (depth) mm. Female gravidity was ascertained visually by clearly discernible abdominal swelling and dilation of the genital opening. After 10 min., the female was gently released, and male and female behaviour was recorded for 12 minutes or until spawning occurred. Pilot studies showed that this length of time was sufficient to capture the full suite of male and female courtship and reproductive behaviours. For males, the frequency of bouts of zigzag courtship behaviour, whereby a male performed an unbroken series of rapid, alternating side-to-side movements [40] and aggressive bites [41] directed at the female, was recorded. For females, the frequency of ‘head-up’ displays; a specific courtship posture performed by gravid females to signal their readiness to spawn [29], following the male to his nest and spawning attempts, whereby a female placed her head at the nest entrance and tried to enter the nest [29], as well as the occurrence of successful spawning, were recorded. Successful spawning was recognised by the female entering the nest of the male, then emerging after a short interval, visibly slimmer after releasing eggs [29]. Once the behaviour recording was complete, the female was removed and measured for Standard Length to the nearest 1 mm. If the female failed to spawn in the mate choice trial, eggs were expressed manually by gently stroking the side of the belly from head to tail with a finger to ensure gravidity. In two cases, females failed to express eggs, and these replicates were discarded and repeated. After spawning, the male’s nest was removed with tweezers and placed in a 90 mm diameter Petri dish. The number of eggs in the nest and the number of polyester threads the nest comprised were counted. After counting, the threads were replaced in the aquarium to allow the male to rebuild his nest, which typically occurred within 24 h, though sometimes in a new location.

Although nest-building conditions were standardised across trials, with a sand substrate, artificial vegetation as refuges, and identical black polyester thread as nest-building material, we acknowledge that unquantified variation in nest features, such as nest entrance size and shape, tidiness of the nest (*sensu* Barber et al. 2001) [30], or location in experimental aquaria, may have contributed to differences in female nest entry and spawning success, representing a limitation of the study.

Males were retained in their aquaria in three further trials in which they were exposed to gravid females of every additional combination of population and ecotype, presented in a randomised order. After completing all four trials, males were measured and not used again in mate choice trials, though they were used to test their response to gravid female nine-spined sticklebacks (see below). All males and females used in mating trials were returned to their loch of origin after completion of trials.

### Male response to female nine-spined sticklebacks

Pilot studies demonstrated that males of the armoured ecotype expressed high levels of aggression towards armourless females. Both the armoured ecotype populations used in the study are sympatric with the nine-spined stickleback (*Pungitius pungitius*), which are potential competitors and egg predators [29]. Females of the three-spined stickleback armourless ecotype superficially resemble female nine-spined sticklebacks (Fig. S1), and we further tested the response of male three-spined sticklebacks to gravid female nine-spined sticklebacks. Here, pairings were made with male three-spined sticklebacks of two ecotypes from four populations with gravid female nine-spined sticklebacks from two populations (Hosta and Reivil). Pairings were made in every possible combination, generating eight pairings in a single complete replicate. Five full replicates of every pairing were conducted for Lochs Hosta and Reivil, six replicates for Loch Fada males and four for Loch Bharpa, yielding a total of 40 male three-spined ×female nine-spined pairings. Male behavioural responses to female nine-spined sticklebacks were made following the same protocol as in mate choice trials. Female nine-spined sticklebacks were returned to their loch of origin at the end of the study.

### In vitro fertilisation

Using the same populations and ecotypes for mate choice trials, we conducted in vitro fertilisations of three-spined stickleback eggs from every population. A batch of eggs was stripped from a female and fertilised with sperm from a male from every combination of population and ecotype. Each unique cross was replicated four times, generating 64 crosses. Gravid females were isolated, and their eggs were stripped onto a 90 mm diameter Petri dish. A batch of 5 eggs was gently separated using a soft artist’s paintbrush and transferred to separate 60 mm diameter Petri dishes containing water from the female’s loch of origin [42]. A small subset of 5 eggs, rather than the entire clutch, was used to minimise density effects on egg survival and development. A male of a predetermined population and ecotype was euthanised via overdose of MS-222 anaesthetic, followed by spinal section, and their testes dissected. Both testes were transferred to a 60 mm diameter Petri dish containing 10 mL of teleost saline (0.5% NaCl, pH neutral; no measurable effect on loch water pH) and disrupted with a dissecting needle to release spermatozoa [43]. A 1 mL subsample of this sperm solution was pipetted over the batch of eggs within 60 s of the eggs contacting water, which were covered and placed in an incubator (WhitePython, Essex, UK) at 17 °C for 30 min. Fertilisation was checked by inspection under a dissecting microscope. The fertilised embryos were washed in water from the female’s loch of origin and photographed alongside a scale bar using a Pentax WG90 digital camera. Egg size was subsequently measured from digital images using ImageJ (v1.54 g) [44]. Fertilised eggs were returned to an egg incubator at 17 °C in filtered (53 μm mesh) loch water from the female egg donor’s loch of origin [42]. Eggs were examined daily, and dead eggs or those showing signs of fungal infection were removed with tweezers. Water in Petri dishes was replaced daily with clean, filtered, aerated loch water. A record was made of embryo survival until hatching. All female three-spined sticklebacks were returned to their loch of origin at the end of the study.

### Data analysis

Data exploration and model validation were undertaken. Model selection was not undertaken, with the exception that if interaction terms did not improve model fit, they were removed. Models were implemented with the R statistical software, version 4.5.0 [45]. Data and R script are available from Smith (2025) [46]. Effect sizes (odds ratios and rate ratios) are reported alongside p-values in results tables.

#### Pre-zygotic reproductive barriers

To test for pre-zygotic reproductive barriers, the probability of females spawning with a given male was modelled using a Bernoulli generalized linear mixed model (GLMM) with a logit link function. Fixed terms in the model were male-female ecotype match (same or different), population match (same or different), and size difference (numerical difference), female length, nest size (measured as number of cotton threads used in nest construction) and male courtship frequency (zigzags). Male identity was included as a random term.

Male nest size, measured as the number of cotton threads used in nest construction, was modelled as a Gamma GLMM with male ecotype as a fixed term and male identity as a random term. Male courtship, measured as the frequency of zigzags, and aggression, measured as the frequency of bites directed at females, were modelled as negative binomial GLMMs with a log link function. Fixed terms in the model were male and female ecotype and their interaction. Male identity was included as a random term. Male courtship and aggression towards gravid female nine-spined sticklebacks were modelled as negative binomial GLMMs with a log link function. Fixed terms in the model were male ecotype and female size. The frequency of female head-up displays, follows to the nest, and spawning attempts was modelled with a negative binomial GLMM with a log link function. Fixed terms in the model were male-female ecotype match (same or different), male courtship frequency (zigzags) and male aggression frequency (bites). Male identity was included as a random term.

#### Post-mating pre- and post-zygotic reproductive barriers

Data for the number of three-spined stickleback eggs that were fertilised and that hatched derived from in vitro fertilisation proved strongly negatively skewed and, consequently, were modelled using a Bernoulli GLM. Fixed terms in both models were ecotype (male and female, either the same or different), population of origin (same or different), female length and mean size of eggs in the clutch.

## Results

### Pre-zygotic reproductive barriers

There were 27 spawnings, representing 34% of all pairings. Of the 16 possible combinations of male-female populations and ecotypes, spawning did not occur in 6 (38%). There was no significant difference in Standard Length ±sd among samples for males (*F*_3,16_ = 0.23, *p* = 0.872); Bharpa = 40.0 ± 1.41 mm, Fada = 40.0 ± 3.16 mm, Hosta = 41.2 ± 2.39 mm, Reivil = 40.8 ± 3.35 mm, or females (*F*_3,76_ = 2.37, *p* = 0.08); Bharpa = 40.2 ± 3.41 mm, Fada = 38.0 ± 3.93 mm, Hosta = 41.8 ± 5.61 mm, Reivil = 39.0 ± 5.48 mm. The mean time to spawning in the study was 6 min 15 s (sd 3 min 5 s).

**Fig. 2:**
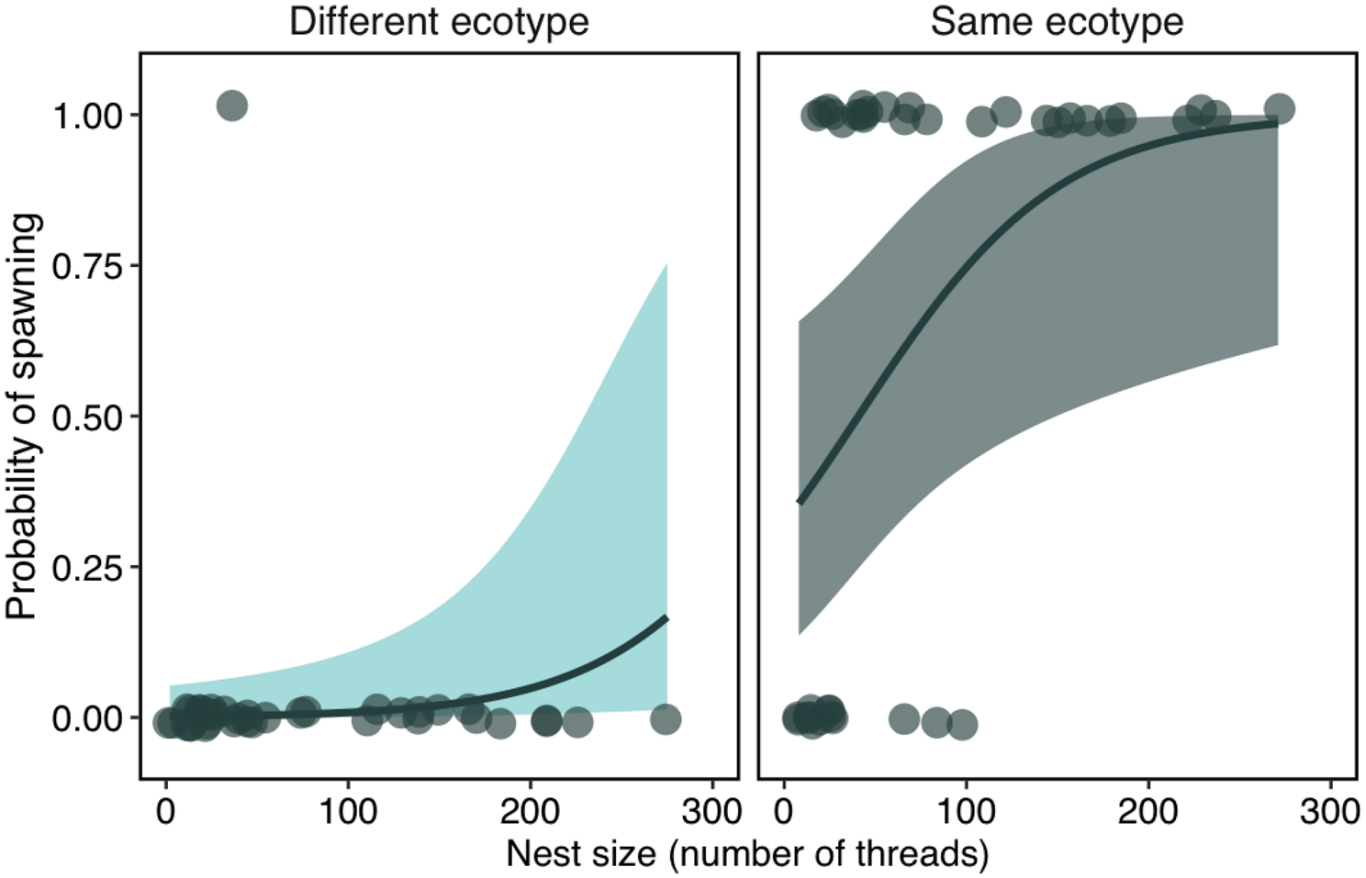
Results of a Bernoulli GLMM analysis showing the relationship between the probability of spawning by female three-spined sticklebacks in the nests of males of the same or a different ecotype as a function of nest size, measured as the number of polyester threads used in nest construction. The mean fitted model is the solid line, the shaded area is the 95% confidence interval. Raw data are shown as dots and are ‘jittered’ to prevent overplotting

**Fig. 3:**
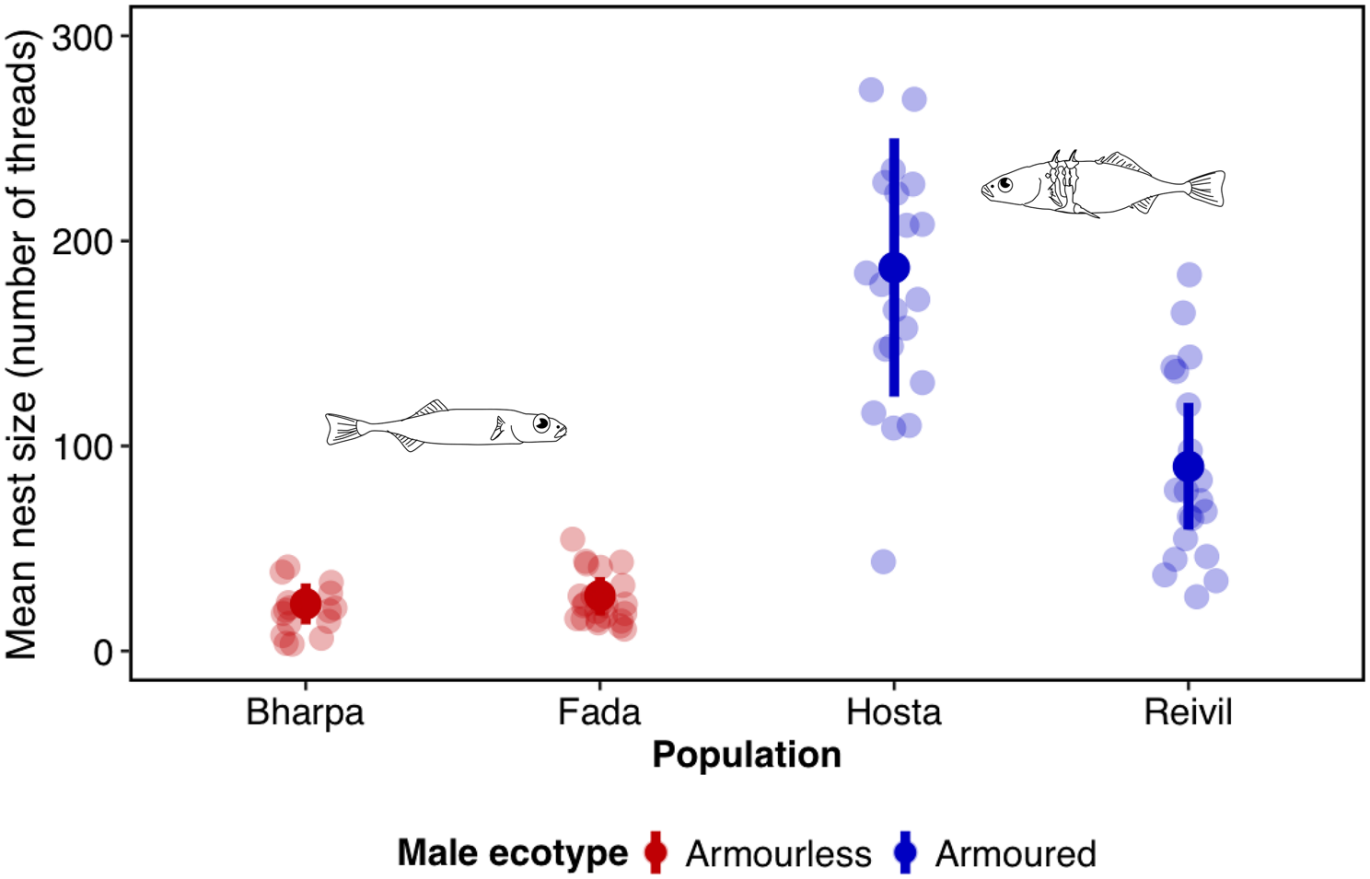
Results of a gamma GLMM analysis showing the relationship between predicted mean nest size, measured as the number of polyester threads used in nest construction, for males from four populations (Lochs Bharpa, Fada, Hosta, Reivil) belonging to two ecotypes (armourless, armoured). Solid dots are the predicted mean nest size, error bars are 95% confidence intervals, and shaded dots are raw data

A shared ecotype was a significant predictor of the probability that a pair of three-spined sticklebacks would spawn (Table 3, Fig. 2). There was also a positive association between nest size and probability of spawning (Fig. 2), though with a small effect size (Table 3). The nest size of armoured males was significantly larger than that of armourless males (Table 4, Fig. 3, Fig. S2). Unusually, one armourless male from Loch Bharpa built a nest amongst vegetation (Fig. S3), in which only a Loch Bharpa female attempted to enter but failed to spawn (Video S1). There was no significant effect of ecotype on the frequency of female head-up displays (Table 5A), follows to the nest (Table 5B) or spawning attempts (Table 5C), though there was a significant positive effect of male courtship frequency on spawning attempts, and a negative effect of male aggression frequency on female follows to the nest and spawning attempts (Table 5), though in each case with low rate ratios.

**Table 3:** Results of a Bernoulli generalised linear mixed model for the probability of spawning for female three-spined sticklebacks from four populations and two ecotypes based on 80 mating trials. Marginal R^2^ = 0.73. OR is the odds ratio

fixed effects	estimate	s.e.	z	p	OR
intercept	−0.14	3.44	−0.04	0.968	0.87
ecotype difference_(same)_	5.89	1.71	3.45	**0.001**	361.2
population difference_(same)_	0.42	0.86	0.49	0.625	1.52
length difference	0.03	0.13	0.19	0.846	1.03
nest size	0.02	0.01	2.28	**0.023**	1.02
female length	−0.15	0.09	−1.63	0.102	0.86
courtship frequency	−0.04	0.04	−1.00	0.317	0.96
**random effect**	**s.d.**	*n*			
male ID	< 0.001	20

**Table 4:** Results of a gamma generalised linear mixed model for the nest size of male three-spined sticklebacks from two ecotypes in 80 mating trials. Marginal R^2^ = 0.70. RR is the rate ratio

fixed effects	estimate	s.e.	z	p	RR
intercept	3.07	0.15	20.85	< 0.001	21.5
male ecotype_(armoured)_	1.72	0.21	8.29	** < 0.001**	5.61
**random effect**	**s.d.**	*n*			
male ID	0.17	20

**Table 5:** Results of negative binomial generalised linear mixed models for female three-spined stickleback behaviour towards male three-spined sticklebacks in 80 mating trials. Head-up display: marginal R^2^ = 0.11, follow: marginal R^2^ = 0.43, follow: spawning attempt R^2^ = 0.47. RR is the rate ratio

Fixed effects	(A) head-up display				(B) follow						C) spawning attempt				
	Estimate	s.e.	z	p	RR	estimate	s.e.	z	p	RR	estimate	s.e.	z	p	RR
intercept	0.97	0.31	3.12	0.002	2.65	0.80	0.41	1.95	0.051	2.23	0.32	0.46	0.69	0.488	1.38
ecotype difference_(same)_	0.12	0.24	0.49	0.627	1.12	0.27	0.32	0.84	0.401	1.31	0.587	0.37	1.59	0.112	1.80
courtship frequency	0.01	0.01	1.21	0.226	1.01	0.02	0.01	1.92	0.055	1.02	0.03	0.01	2.22	**0.027**	1.03
aggression frequency	−0.02	0.02	−1.23	0.220	0.98	−0.09	0.03	−3.14	**0.002**	0.92	−0.11	0.03	−3.42	** < 0.001**	0.89
**random effect**	**s.d.**	*n*				**s.d.**	*n*				**s.d.**	*n*			
male ID	< 0.001	20				< 0.001	20				< 0.001	20			

**Fig. 4:**
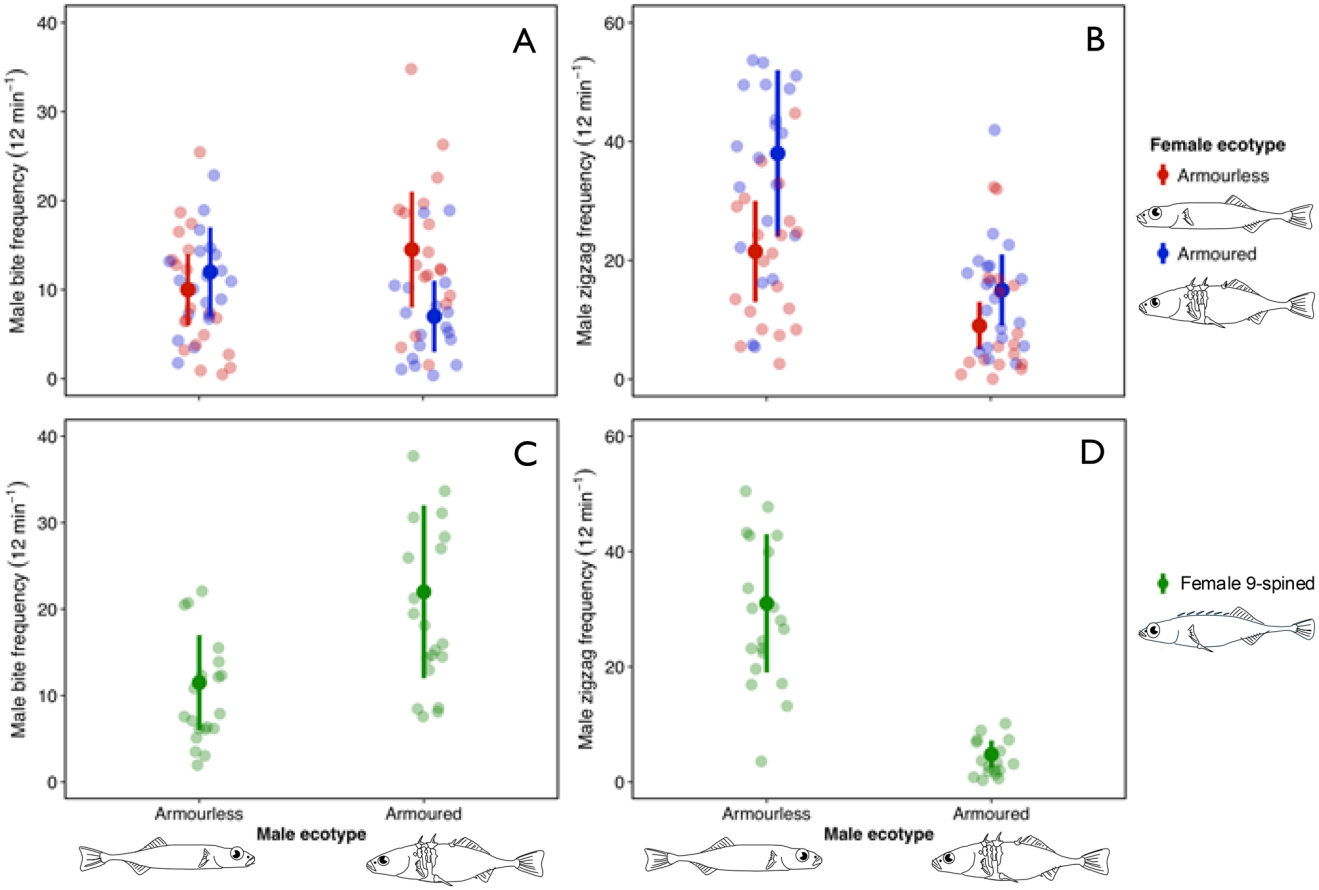
Results of a negative binomial GLMM analysis showing the relationship between male aggression and courtship behaviour for males of two ecotypes (armourless, armoured). (**A**) Male aggression, measured as bite frequency, directed at gravid armourless and armoured female three-spined sticklebacks. (**B**) Male courtship, measured as zigzag frequency, directed at gravid armourless and armoured female three-spined sticklebacks. (**C**) Male aggression, measured as bite frequency, directed at gravid female nine-spined sticklebacks. (**D**) Male courtship, measured as zigzag frequency, directed at gravid female nine-spined sticklebacks. Solid dots are the predicted mean behaviour frequency, error bars are 95% confidence intervals, and shaded dots are raw data

Armoured male three-spined sticklebacks directed a significantly higher frequency of aggression towards armourless than armoured female three-spined sticklebacks (Table 6A, Fig. 4A). Armourless males demonstrated no significant difference in aggression frequency towards armoured and armourless female three-spined sticklebacks (Table 6A, Fig. 4A). Armoured male three-spined sticklebacks showed a significantly greater frequency of aggression towards gravid female nine-spined sticklebacks, regardless of the female population of origin, compared to armourless males (Table 7A, Fig. 4B).

**Table 6:** Results of negative binomial generalised linear mixed models for male three-spined stickleback behaviour towards female three-spined sticklebacks in 80 mating trials. Aggression: marginal R^2^ = 0.26, courtship: marginal R^2^ = 0.59. RR is the rate ratio

fixed effects	(A) aggression (bites)					(B) courtship (zigzags)				
	estimate	s.e.	z	p	RR	estimate	s.e.	z	p	RR
intercept	2.19	0.20	11.13	< 0.001	8.96	2.98	0.15	19.63	< 0.001	19.6
male ecotype_(armoured)_	0.37	0.23	1.58	0.115	1.45	−0.94	0.16	−5.74	** < 0.001**	0.39
female ecotype_(armoured)_	0.21	0.23	0.90	0.367	1.23	0.58	0.14	4.19	** < 0.001**	1.78
length difference	0.01	0.03	0.21	0.835	1.00	0.002	0.02	0.09	0.926	1.00
male : female ecotype_(armoured)_	−1.00	0.35	−2.89	**0.004**	0.37	-	-	-	-	
**random effect**	**s.d.**	*n*				**s.d.**	*n*			
male ID	0.08	20				0.17	20

**Table 7:** Results of negative binomial generalised linear mixed models for the behaviour of male three- spined sticklebacks towards female nine-spined sticklebacks across 40 trials. Aggression: marginal R^2^ = 0.15, courtship: marginal R^2^ = 0.71. RR is the rate ratio

fixed effects	(A) aggression (bites)					(B) courtship (zigzags)				
	estimate	s.e.	z	p	RR	estimate	s.e.	z	p	RR
intercept	2.23	0.64	3.47	0.001	9.25	2.20	0.67	3.29	0.001	8.99
male ecotype_(armoured)_	0.641	0.19	3.45	**0.001**	1.90	−1.94	0.23	−8.40	** < 0.001**	0.14
female length	0.002	0.02	0.11	0.915	1.00	0.03	0.02	1.77	0.077	1.03
**random effect**	**s.d.**	*n*				**s.d.**	*n*			
male ID	0.28	20				0.18	20			

The frequency of courtship by armoured and armourless males towards armoured female three-spined sticklebacks was significantly greater than towards armourless females (Table 6B, Fig. 4C). Armourless males courted both armoured and armourless females at a higher frequency than armoured males (Table 6B, Fig. 4C). Armoured male three-spined sticklebacks exhibited a significantly lower frequency of courtship towards gravid female nine-spined sticklebacks, irrespective of the female population of origin, compared to armourless males (Table 7B, Fig. 4D). There was no diminution in male courtship among consecutive mating trials (*F*_3,76_ = 1.02, *p* = 0.387).

### Post-mating pre-zygotic and post-zygotic reproductive barriers

From a total of 320 eggs, 279 (87%) were successfully fertilised; of those fertilised, 264 (95%) survived their first 48 h, and 237 (85%) hatched. Of the 64 clutches of eggs, 3 (5%) entire clutches were unfertilised, 4 (6%) suffered 100% mortality, and 31 (48%) showed 100% survival from fertilisation to hatching. We detected no significant effect of ecotype, population, female size or egg size on fertilisation success (Table 8A) or hatching success (Table 8B).

**Table 8:** Results of Bernoulli generalised linear models for the fertilisation and hatching success of three-spined stickleback eggs derived from 64 in vitro fertilisations: fertilisation success R^2^ = 0.13, hatching success R^2^ = 0.02. OR is the odds ratio

fixed effects	(A) fertilisation success					(B) hatching success				
	estimate	s.e.	z	p	OR	estimate	s.e.	z	p	OR
intercept	−6.02	9.81	−0.61	0.540	0.02	−3.83	7.20	−0.53	0.595	0.02
ecotype_(same)_	2.14	1.13	1.90	0.058	8.52	0.51	0.63	0.82	0.413	1.67
population_(same)_	−0.84	1.30	−0.65	0.516	0.43	−0.22	0.72	−0.31	0.758	0.80
female length	−0.05	0.05	−0.98	0.326	0.95	−0.01	0.04	−0.16	0.874	0.99
mean egg size	5.31	5.53	0.96	0.337	0.02	2.32	3.91	0.59	0.553	10.2

## Discussion

While the armoured and armourless ecotypes of the three-spined stickleback on North Uist have evolved in geographic (allopatric) isolation, our results demonstrate a clear pre-zygotic barrier, evident as reduced spawning success between the two ecotypes under no-choice conditions, which are at an early stage of divergence. The mechanism underlying this reduced spawning success could be female mate choice, behavioural interference, mechanical constraints, or a combination thereof; our experimental design does not conclusively distinguish among these mechanisms. However, two barriers that appeared to contribute to reduced spawning success between ecotypes were differences in nest structure and behavioural interference associated with male aggression. The concurrence of these two reproductive barriers between ecotypes appears not to be independent, since both reflect differences in morphology and behaviour that arise from adaptation to two discrete environments. The coincidence of these two reproductive barriers, termed ‘coupling’ (*sensu* Butlin & Smajda 2018) [47], may operate to further reinforce sexual isolation between the ecotypes.

Nests are built by various vertebrate and invertebrate species, and while their primary purpose is to provide a space for eggs and offspring to develop, they can also signal quality to potential mates [48]. As a result, nest construction is potentially a target of selection, both in the context of natural selection, by enhancing offspring survival, and sexual selection, as an indicator of mate quality [49]. The nest of the three-spined stickleback is a complex structure that functions in spawning and parental care of the offspring [29]. It is built by the male and is at least partially under genetic control [50]. Here, we demonstrate that certain features of nest structure can limit gene flow among ecotypes where they occur together, albeit in one direction. In this study, a possible reason for the failure of armoured females to enter the nests of armourless males was the difference in nest size between ecotypes; armourless males built nests 57% smaller than armoured males, which armourless females were able to use, but which more robust armoured females appeared unable to enter despite attempting to do so. Other nest features, in addition to size, may also have been important. For example, nest entrance shape, other aspects of nest construction, or nest location may have played roles. Nest materials were standardised experimentally by providing strands of black polyester thread, though some males did incorporate small pieces of vegetation that they extracted from the natural sand substrate with which they were provided, potentially influencing female spawning decisions. Water chemistry may also have affected nest construction. Thus, increased turbidity resulted in the construction of nests that were approximately 30% smaller than in clear water [51]. There is also evidence for locally-adapted *spiggin*, the adhesive that males produce to stick their nests together, that possesses different functional properties in contrasting physicochemical environments [52]. Despite these potential differences between nests built by the two ecotypes, nest size was by far the most conspicuous difference, and we offer the effect of nest size as a working hypothesis to explain the failure of armoured females to spawn with unarmoured males. An experimental approach to this question will be explored in future research. In a pilot study, we successfully switched the nests of armoured and armourless males, which were adopted by males, at least in the short term. This approach offers the possibility of separating male ecotype and nest architecture to identify the primary driver of female spawning biases.

While armourless males vigorously courted armoured females, which readily followed males to their nests, we observed no cases where they entered the nest of an armourless male, though the converse did occur, with an armourless (Fada) female spawning in the nest of an armoured (Reivil) male. In a study of two sympatric three-spined ecotypes in Lake Thingvallavatn, Iceland, Ólafsdóttir et al. (2006) [53] also identified nest structure and location as potential pre-zygotic barriers to gene flow. These ecotypes show restricted gene flow and appear to be reproductively isolated [53]. Similarly, Dean et al. (2021) [54] investigated mating preferences between low and completely-plated ecotypes on North Uist and detected a greater probability of spawning between members of the same ecotype, which they attributed to differences between ecotypes in the choice of nest substrate. Thus, adaptive divergence in nest characteristics that are tailored to local environments may create mating barriers, particularly if female nest preferences co-evolve with local nest characteristics, a subject that has hitherto received little attention [50].

While armoured females failed to spawn with armourless males, possibly due to nest characteristics, armourless females, with a single exception, also failed to spawn in the nests of armoured males, possibly due to the latter’s aggression towards them; an aggressive response to gravid females has been recognised as resulting in low reproductive success in male three-spined sticklebacks [55], but also in other taxa. Notably, armoured males showed aggression to armourless female three-spined sticklebacks at a similar rate to that towards nine-spined sticklebacks, with which they are sympatric. Aggression between three- and nine-spined sticklebacks is commonplace under natural conditions as well as in the lab [56]. Armourless males showed no difference in aggression frequency between armoured and armourless females. They also courted gravid armoured and armourless three- and nine-spined females at similar rates. In contrast, armoured males courted gravid female nine-spined sticklebacks occasionally, but at a substantially lower frequency than did armourless males. Armourless female three-spined sticklebacks are relatively slim-bodied and dull-coloured, even when gravid, and lack a conspicuously distended belly, a trait that elicits elevated courtship by male three-spined sticklebacks [57]. They also superficially resemble nine-spined sticklebacks; stickleback species may share perceptual biases, as both species respond to similar visual cues [29].

Male courtship likely serves several functions. It indicates the presence of a reproductive male, provides an opportunity for mate choice, ensures the eggs are laid in the correct location for their subsequent care, and guarantees the male mates with a female of his own species [58]. To achieve successful courtship and mating while simultaneously defending a nest and territory, a male must switch between these actions in response to appropriate stimuli for each behaviour [59]. However, the relevant stimuli for nest defence and mate finding may have overlapping stimulus properties, with the potential to release both types of behaviour [29]. For example, while gravid female three-spined sticklebacks represent a risk to offspring, as well as a spawning opportunity, non-gravid females primarily represent potential predators of eggs to nest-guarding males [60]. An outcome is that when gravid armourless females are presented to armoured males, their less conspicuously gravid appearance may fail to elicit courtship reliably, instead stimulating aggression. This finding matches the predictions of a theoretical model [61] for the expression of intersexual aggression, with males carrying an internal template against which they match relevant cues presented by the phenotype of an actor to their territory.

While armoured male aggression towards armourless females seemingly contributed to spawning failure, other mechanisms preventing or inhibiting armourless females from spawning with armoured males may also operate. Notably, armourless females performed the full repertoire of courtship behaviours (head-up displays, nest follows, spawning attempts) regardless of male ecotype, which suggests that armourless females may have rejected spawning with armoured males based on visual or olfactory cues, or nest characteristics, as discussed above. These mechanisms are not mutually exclusive and may interact. The no-choice experimental design highlights realised spawning failure but cannot fully distinguish female choice from male-driven barriers, supporting the possibility of multiple contributing factors. Future experiments manipulating specific cues would clarify their relative roles.

Our results show that armoured males direct significantly higher aggression (measured as bite frequency) specifically toward armourless females and gravid female nine-spined sticklebacks compared to armoured conspecific females, with no equivalent ecotype-specific difference observed in armourless males. However, it remains unclear whether this elevated aggression reflects a general behavioural tendency, with armoured males inherently more aggressive across contexts, or a context-dependent response triggered by perceptual mismatches in female phenotype. Previous research has shown the opposite pattern [62–64], with higher lateral plate number linked to lower male aggressiveness, and direct tests of armoured male responses to additional stimuli, such as rival males or egg predators, are needed to distinguish these alternatives.

Three-spined stickleback armoured/armourless ecotypes on North Uist have evolved in allopatry. Thus, premating barriers have evolved after habitat isolation, and geographic isolation is the most prominent barrier to the hybridisation of the ecotypes we investigated. However, these ecotypes do show secondary contact in at least one location on North Uist. Here, an acidic peat loch, Loch nan Geireann (57.600521, −7.182698), debouches into coastal freshwater Loch na Ciste (57.598345, −7.177055). Loch nan Geireann supports a population of armourless three-spined sticklebacks, some of which escape into Loch na Ciste through a culvert under a road, where they are confined to a pool at the Loch’s western end. Loch na Ciste, although freshwater and 3 m above sea level, connects to a sea loch through a narrow cataract. At high tide, the loch is connected to the sea loch, and in spring, anadromous, completely-plated three-spined sticklebacks enter the loch to spawn in freshwater. An outcome is that three ecotypes co-occur and spawn in the Loch: completely-plated migratory, low-plated resident (comparable to the ‘armoured’ ecotypes from Lochs Hosta and Reivil) and the displaced ‘armourless’ ecotype from Loch nan Geireann. Dean et al. (2024) [65] conducted a genomic analysis of these populations, demonstrating that freshwater low-plated (what they erroneously termed ‘saltwater resident’) and anadromous completely-plated fish hybridise, creating a partially-plated hybrid, while they detected no evidence of introgression between either the low or completely-plated ecotypes and the armourless ecotype. Hybridisation between low or completely-plated three-spined sticklebacks is unremarkable; it was described over a century ago [66] and is common in the lower reaches of rivers and estuaries around Scotland [15, 67]. However, the failure of the armoured low-plated Loch na Ciste and armourless Loch nan Geireann three-spined sticklebacks to hybridise in nature lends support to our aquarium observations, though whether this barrier is maintained through sexual isolation or from some other mechanisms is unclear. Thus, while our laboratory experiments provide evidence of strong pre-zygotic barriers to mating between armoured and armourless ecotypes under controlled no-choice conditions, these findings may not fully capture the complexities of mating barriers in natural secondary contact scenarios, such as the overlap observed in certain North Uist lochs. Broader evolutionary implications, including the potential reinforcement of isolation in the wild, therefore remain speculative and warrant further field-based investigation.

We detected no post-mating pre-zygotic or post-zygotic reproductive barriers to hybridisation between armoured and armourless ecotypes. This analysis was based on short-term outcomes measured in terms of fertilisation success and survival to hatching, representing ‘intrinsic’ barriers that were measured in the benign, stable, but replicable environment of a Petri dish and incubator. This outcome mirrors comparable studies on hybrids between three-spined stickleback benthic–limnetic ecotypes from the Strait of Georgia region of British Columbia, for which intrinsic barriers are also weak or absent [12]. However, ‘extrinsic’ barriers, which must be observed in the appropriate ecological setting, may better reflect hybrid performance under natural conditions, with ecologically-mediated natural selection removing low-fitness hybrids [68]. Thus, Thompson et al. (2022) [69] showed contrasting outcomes for hybrids from crosses between three-spined stickleback benthic-limnetic ecotypes reared in aquaria and semi-natural experimental ponds, demonstrating the significance of ecologically-mediated selection against hybrids that represent maladaptive phenotypes under natural conditions. Consequently, it may be premature to assume the absence of extrinsic post-mating pre- or post-zygotic barriers without more fully dissecting hybrid performance [6].

While the differences in nest structure and male aggression observed here represent striking adaptations associated with extreme armour reduction in North Uist three-spined stickleback populations, it remains uncertain whether these patterns are general across other armour-reduced populations worldwide; such highly derived ‘armourless’ ecotypes (lacking functional plates, spines, and pelvic girdle) are globally rare and we are unaware of comparable studies to this one. Future comparative research on populations from British Columbia and Alaska, with varying degrees of armour loss, is needed to determine the broader applicability of these traits as reproductive barriers. Although our study demonstrates clear ecotype-specific differences in nest architecture and male aggression that likely function as pre-zygotic barriers, we have not distinguished whether these traits reflect fixed genetic divergence or environmentally induced phenotypic plasticity; given the pronounced habitat differences (particularly pH and ion concentrations) between peat and *machair* lochs, and the known plasticity of behavioural and morphological traits in three-spined sticklebacks [70], it remains an open question whether these differences would persist under common-garden rearing conditions, and reciprocal transplant or common-environment experiments will be required to resolve the relative contributions of genetic adaptation and plasticity.

A caveat to this study is that, in mate choice trials, females were transferred from the water of their loch of origin into the water of the male’s loch of origin after a period of acclimation. If a change in pH disrupted female propensity to spawn, this could explain our findings regarding a failure of females to mate with males of a different ecotype. However, several pieces of evidence argue against this conclusion. The first is that spawning did occur between ecotypes, notably with an armourless Loch Fada female spawning in the nest of an armoured Loch Reivil male; thus, the change in pH did not inhibit spawning. In addition, the pH between populations within ecotypes was not identical; that between Hosta and Reivil differed by 0.9, suggesting females coped with pH change and were capable of spawning readily. Indeed, the fact that we detected no population effects in our analysis mitigates against water chemistry as the primary driver of spawning failure by females. Future common-garden experiments rearing ecotypes in standardised water conditions could further confirm whether pH directly influences spawning propensity or nest architecture. Finally, females expressed their full repertoire of spawning behaviours, including head-up displays, following males to their nest, and spawning attempts, irrespective of ecotype treatments, indicating that it was not the failure of females to perform spawning behaviour that led to low spawning rates, but rather the inability of armoured females to enter the nests of armourless males, and the high frequency of aggression from armoured males directed at armourless females that resulted in the failure of females to spawn.

## Conclusion

Our study on three-spined stickleback ecotypes on North Uist indicates that distinct pre-zygotic reproductive barriers, reinforced by sexual isolation, characterise early-stage speciation. The geographic isolation driven by environmental gradients, particularly pH variation, aligns with prior research, reinforcing the role of ecological factors in shaping reproductive isolation. This study uniquely highlights a pre-mating barrier linked to nest construction in a globally rare armourless stickleback ecotype, contrasting with previous studies emphasising size-assortative mating, nuptial colouration, or habitat preferences as reproductive barriers. Additionally, armoured males exhibit asymmetric aggression towards armourless females, thereby inhibiting female mating attempts and representing a behavioural barrier not previously recognised in the species. Our findings represent an example of the coupling of barrier effects and underscore the importance of integrating ecological and behavioural data to understand the mechanisms driving speciation.

## Electronic supplementary material

Below is the link to the electronic supplementary material.

Supplementary Material 1

Supplementary Material 2. **Video S1** Armourless male three-spined stickleback from Loch Bharpa, North Uist, courting and leading a female from the same population to a nest he has built on the top of an artificial aquarium plant.

## Data Availability

Data and R script are available in the FigShare Digital Repository.
