# Supplementary material for: Nest architecture and male aggression drive sexual isolation between three-spined stickleback ecotypes
Source: Zoological Lett. 2026 Feb 12;12:5. doi: 10.1186/s40851-026-00263-w (PMC13055007; doi:10.1186/s40851-026-00263-w)

Fig. S1 Stickleback species and ecotypes used in the study. (A) Armoured (low-plated) male three-spined stickleback (*Gasterosteus aculeatus*); (B) armoured (low-plated) gravid female three-spined stickleback; (C) armourless male three-spined stickleback; (D) armourless gravid female three-spined stickleback; (E) gravid female nine-spined stickleback (*Pungitius pungitius*). Scale bars are 5 mm.

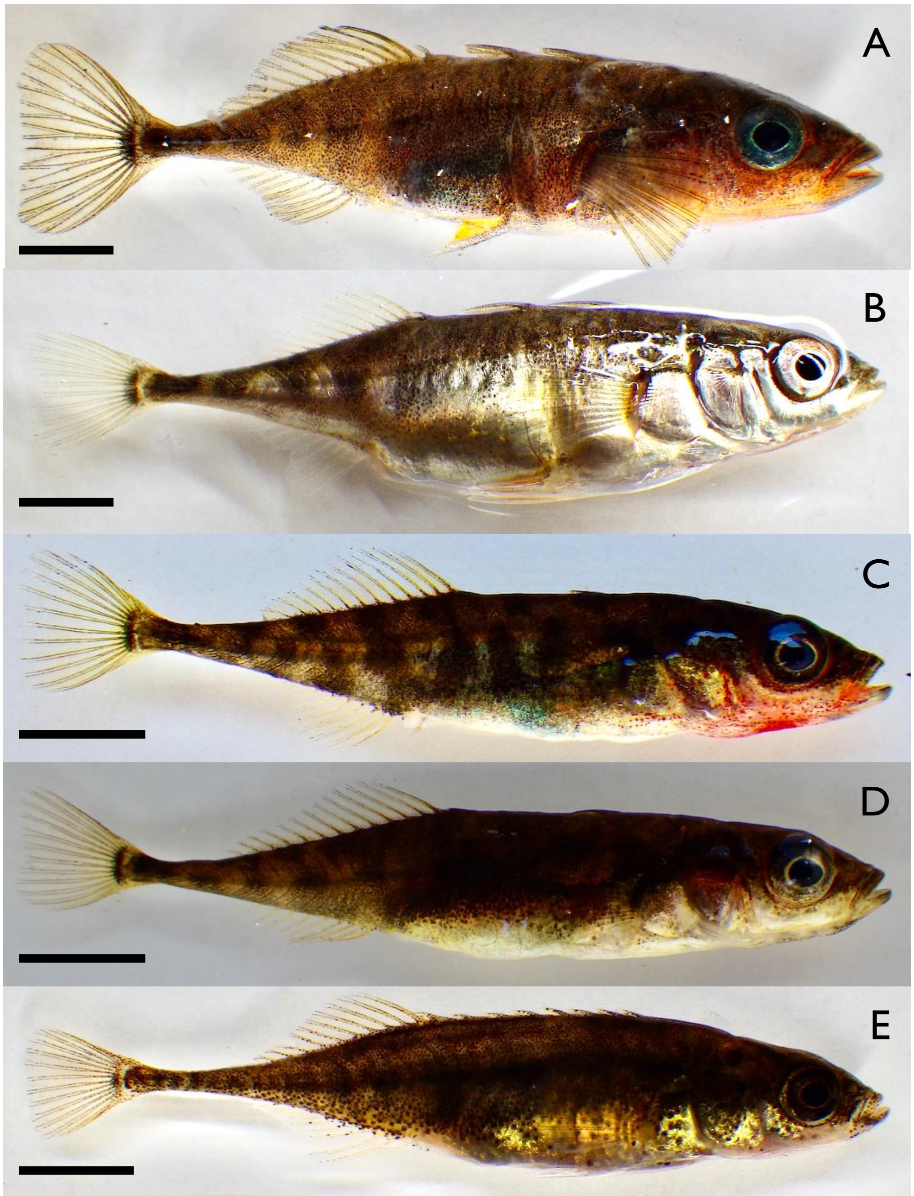

Fig. S2 Examples of nests built by: (A) armoured (low-plated) male three-spined stickleback; (B) armourless male three-spined stickleback. Squares are 5 mm.

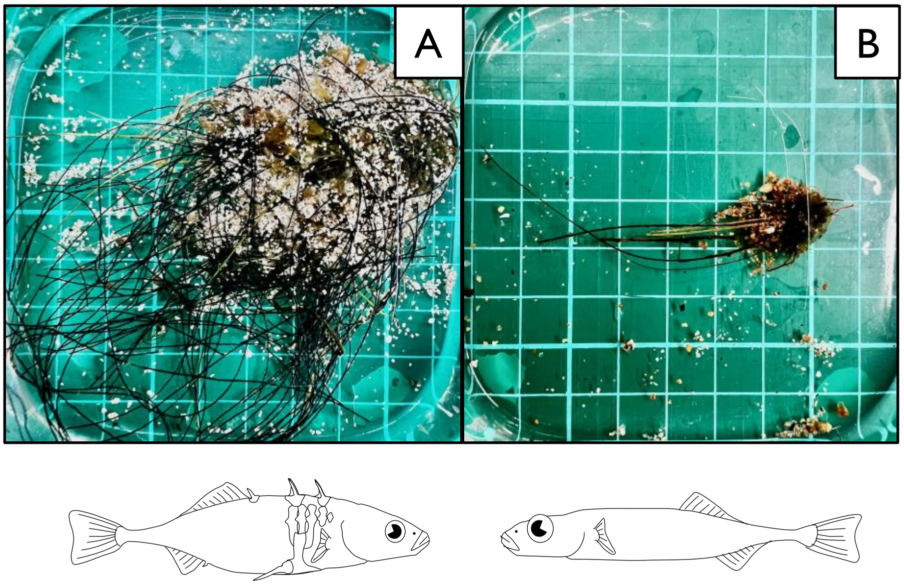

Fig. S3 The nest built by an armourless male three-spined stickleback from Loch Bharpa, North Uist. The nest is built on the top of a 70 mm tall artificial plastic plant. The entrance to the nest is indicated by an arrow. Squares are 5 mm.

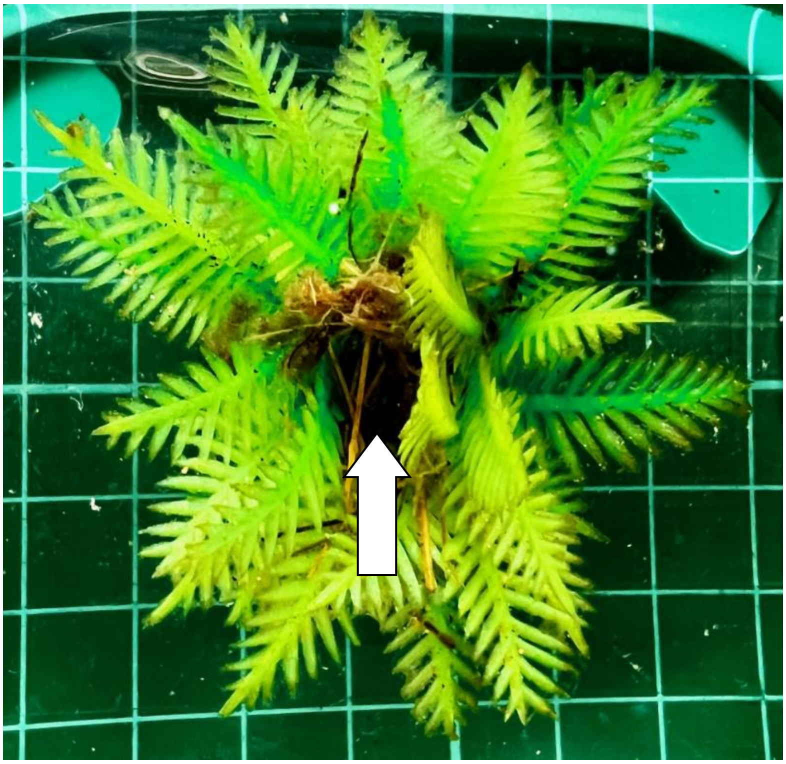

Supplement: Supplementary file 1 — Supplementary Material 1 [file 40851_2026_263_MOESM1_ESM.pdf]
